# Gut microbiota and host genetics contribute to the phenotypic variation of digestive and feed efficiency traits in growing pigs fed a conventional and a high fiber diet

**DOI:** 10.1186/s12711-022-00742-6

**Published:** 2022-07-27

**Authors:** Vanille Déru, Francesco Tiezzi, Céline Carillier-Jacquin, Benoit Blanchet, Laurent Cauquil, Olivier Zemb, Alban Bouquet, Christian Maltecca, Hélène Gilbert

**Affiliations:** 1 GenPhySE, INRAE, ENVT, Université de Toulouse, 31320 Castanet Tolosan, France; 2 France Génétique Porc, 35651 Le Rheu Cedex, France; 3 Department of Animal Science, North Carolina State University, Raleigh, NC USA; 4 Department of Agriculture, Food, Environment and Forestry, University of Florence, 50144 Florence, Italy; 5 UE3P, INRAE, Domaine de la Prise, 35590 Saint-Gilles, France; 6 IFIP-Institut du Porc, 35651 Le Rheu Cedex, France

## Abstract

**Background:**

Breeding pigs that can efficiently digest alternative diets with increased fiber content is a viable strategy to mitigate the feed cost in pig production. This study aimed at determining the contribution of the gut microbiota and host genetics to the phenotypic variability of digestive efficiency (DE) traits, such as digestibility coefficients of energy, organic matter and nitrogen, feed efficiency (FE) traits (feed conversion ratio and residual feed intake) and growth traits (average daily gain and daily feed intake). Data were available for 791 pigs fed a conventional diet and 735 of their full-sibs fed a high-fiber diet. Fecal samples were collected at 16 weeks of age to sequence the V3–V4 regions of the 16S ribosomal RNA gene and predict DE with near-infrared spectrometry. The proportions of phenotypic variance explained by the microbiota (microbiability) were estimated under three OTU filtering scenarios. Then, microbiability and heritability were estimated independently (models Micro and Gen) and jointly (model Micro+Gen) using a Bayesian approach for all traits. Breeding values were estimated in models Gen and Micro+Gen.

**Results:**

Differences in microbiability estimates were significant between the two extreme filtering scenarios (14,366 and 803 OTU) within diets, but only for all DE. With the intermediate filtering scenario (2399 OTU) and for DE, microbiability was higher (> 0.44) than heritability (< 0.32) under both diets. For two of the DE traits, microbiability was significantly higher under the high-fiber diet (0.67 ± 0.06 and 0.68 ± 0.06) than under the conventional diet (0.44 ± 0.06). For growth and FE, heritability was higher (from 0.26 ± 0.06 to 0.44 ± 0.07) than microbiability (from 0.17 ± 0.05 to 0.35 ± 0.06). Microbiability and heritability estimates obtained with the Micro+Gen model did not significantly differ from those with the Micro and Gen models for all traits. Finally, based on their estimated breeding values, pigs ranked differently between the Gen and Micro+Gen models, only for the DE traits under both diets.

**Conclusions:**

The microbiota explained a significant proportion of the phenotypic variance of the DE traits, which was even larger than that explained by the host genetics. Thus, the use of microbiota information could improve the selection of DE traits, and to a lesser extent, of growth and FE traits. In addition, our results show that, at least for DE traits, filtering OTU is an important step and influences the microbiability.

**Supplementary Information:**

The online version contains supplementary material available at 10.1186/s12711-022-00742-6.

## Background

Currently in modern capital-intensive systems of pig production, feed represents between 60 and 70% of the total cost of pork production [1], and to limit this cost, feed efficiency (FE) remains the main selection objective. To reduce feed costs and the amount of agricultural land dedicated to the production of animal feed, one option is to feed pigs with by-products from the agri-food and biofuel industry. However, by-products have an increased fiber content and lower energy than conventional feed resources and these have a negative impact on FE and growth performances [2–4]. Recent research has suggested that improving digestive efficiency (DE) could in turn improve FE when such alternative resources are used, because of its sizable genetic variability, particularly when pigs are fed diets with an increased fiber content [5]. Digestive efficiency is influenced by host genetics [5], but other factors, such as the gut microbiota composition, could also significantly affect this trait. The fermentation activity by the microbiota in the large intestine directly impacts the digestibility of nutrients [6, 7], especially when the diet contains dietary fibers, which cannot be assimilated without fermentation [8]. Moreover, a recent study demonstrated the existence of genetic correlations between DE traits and some heritable traits of the fecal microbiota [9].

Microbiability ($m^{2}$) is the proportion of phenotypic variance explained by the microbiota [10] and it can be used to determine the impact of microbiota composition on traits of interest in growing pigs. As reported by Ross et al. [11], to estimate the $m^{2}$ it is necessary to construct a microbial covariance matrix, which can then be coupled with a standard linear mixed model as used to predict breeding values based on pedigree or genomic covariance. Based on porcine fecal samples, $m^{2}$ estimates of ~ 0.20 for feed conversion ratio (FCR), daily feed intake (DFI), and average daily gain (ADG and similar heritability ($h^{2}$) and $m^{2}$ estimates for FCR and DFI have been reported [12, 13]. Another study found an even higher $m^{2}$ estimate (~ 0.45) for residual feed intake (RFI) [14]. Given the difficulty to record DE traits, to date, only one study including a limited number of pigs has shown that fecal microbiota can explain a significant part of their phenotypic variation [15]. The authors of that study found high $m^{2}$ estimates (> 0.53 ± 0.24) for the digestibility coefficients (DC) of dry matter, organic matter, crude protein, crude fat, crude fiber and non-starch polysaccharides. Taken together, these results suggest that fecal microbiota could explain a significant fraction of the phenotypic variance of DE traits. In addition, since DE is also under the control of host genetics [5], it is interesting to estimate the contribution of the genetic and microbiota effects separately and jointly on DE traits. Moreover, as the dietary fiber content affects the composition of gut microbiota [16, 17], microbiota-by-diet interactions may exist, in the same way as the genetic-by-diet interactions that have been demonstrated for DE [5], thus the effect of diet on $m^{2}$ should be investigated.

Preprocessing of microbial sequencing data is one of the main challenges when comparing results from different studies that investigate the contribution of the microbiota to host phenotypic variation. In particular, the filtering criteria applied to the operational taxonomic units (OTU) that are used in subsequent analyses varies greatly between studies. For example, some authors choose to retain the OTU that are present in more than 50% of the samples [18] while others in at least 5% of the samples [15]. Thus, in most cases, $m^{2}$ estimates cannot be compared between studies.

Here, our aim was to estimate the proportion of phenotypic variance for FE and DE traits explained by the microbiota and by the host genetics separately and jointly, in a Large White (LW) pig population fed a conventional (CO) or a less digestible diet with an increased fiber content (HF). First, we evaluated the impact of different OTU filtering criteria on the $m^{2}$ estimates. Second, we compared the proportion of variance explained by the microbiota and the host genetics for FE and DE traits. Third, we evaluated the impact of including both genomic and microbiota information in a linear mixed model on estimated genomic breeding values (GEBV) and estimated microbiota values (EMV). Finally, we quantified the relevance of the interactions between diet and genetics/microbiota and assessed the stability of the estimated genetic and microbiota parameters to the two CO and HF diets.

## Methods

The study was conducted in accordance with the French legislation on animal experimentation and ethics. The certificate of Authorization to Experiment on Living Animals was issued by the Ministry of Higher Education, Research and Innovation to conduct this experiment under reference number 2017011010237883 at INRAE UE3P—France Génétique Porc phenotyping station (UE3P, INRAE, 2018. Unité expérimentale Physiologie et Phénotypage des Porcs, France, 10.15454/1.5573932732039927E12).

### Experimental design

In the current study, we used the same animals as those described in Déru et al. [3]. In total, 1942 purebred Large White (LW) male pigs were reared in 35 consecutive batches between 2017 and 2018 at the INRAE UE3P France Génétique Porc phenotyping station under two dietary conditions. The study was designed to genetically connect the datasets obtained with the two diets, i.e. to facilitate the estimation of genetic covariances between diets in a dataset of limited size, from each pair of full sibs of homogeneous weights, one was fed one diet and the other the alternative diet. All pigs were obtained from 171 sires that were representative of those used in the French Large White collective breeding scheme, and each pair of full-sibs came from a different dam.

Housing conditions and management of pigs were as described in Déru et al. [3]. Upon arrival, each pair of full sibs was separated and allotted in pens of 14 animals. Pigs were raised in post-weaning facilities until 9 weeks of age and fed with a standard two-phase post-weaning dietary sequence. Then, they were moved to the growing–finishing facilities without mixing until they reached slaughter weight (115 kg body weight). One of the siblings was fed the CO diet and the other the HF diet (see their composition in the next section). Each growing–finishing pen contained a single place electronic feeder equipped with a weighing scale (Genstar, Skiold Acemo, Pontivy, France) to record feed intake and individual body weight of the animal at each visit to the feeder. At a body weight of 115 kg, pigs were fasted for 24 h and then transported to the slaughterhouse. Animals were slaughtered in 89 slaughter batches of approximately 19 pigs.

### Diets

During the growing–finishing phase, each of the two sets of pigs was fed a different two-phase dietary sequence. First, a growing diet was distributed, then at 16 weeks of age a 5-day transition was performed, and a finishing diet was provided until the end of the test. The HF diet included both insoluble and soluble dietary fibers. The detailed compositions of the CO and HF diets are provided in Additional file 1: Table S1. Based on their formulation, the diets differed in net energy (NE), with 9.6 MJ/kg for the CO diet and 8.2 MJ/kg for the HF diet for both two-phase dietary sequences. The diets also differed in neutral detergent fiber (NDF), that ranged from 13.90 to 15.12% for the CO diet and from 23.82 to 24.46% for the HF diet for the two-phase dietary sequences. The ratio of digestible lysine to NE was identical in both dietary sequences, and was equal to 0.97–0.99 g/MJ NE in the growing phase and 0.83 g/MJ NE in the finishing phase.

### Recorded traits and sampling

For each animal, ADG and DFI were measured between 35 and 115 kg. ADG was computed as the ratio between body weight gain and number of days on test. The FE traits were FCR and RFI. FCR was calculated as the ratio between DFI and ADG and was expressed in kg/kg. For the two diets, RFI was determined using a single multiple linear regression [19] of DFI on ADG, lean meat percentage and carcass yield recorded at slaughter, and average metabolic body weight as described in Déru et al. [3]. The proportion of pigs that experienced health problems or injury during the test period was the same in the two diet groups, and these were discarded from the analysis. Among the 1942 pigs in the experiment, 1663 had data available for FCR, ADG and DFI, and only 1595 pigs had data for RFI.

DE and fecal microbiota composition were also determined for the animals in this study. A unique fecal sample was collected at 16 weeks of age just before feed transition between the growing and finishing phases to measure DE and to analyze microbiota composition. For each pig, feces were collected in a piping bag and manually homogenized. About 50 g of feces were stored in plastic containers at − 20 °C until further analyses to measure DE traits. Samples were freeze–dried and ground with a grinder (Grindomix GM200, Retsch). DE was computed based on the DC of energy, nitrogen and organic matter predicted using near infrared spectrometry (NIRS) analyses of these samples, as described in detail in Déru et al. [5]. The prediction equations for the DC of organic matter, nitrogen and energy were reliable, with cross-validation R^2^ values higher than 0.89 [20]. In total, data for DC were available for 1242 pigs among which 654 were fed a CO diet and 588 a HF diet. Another fraction of the feces samples was used to assess the microbiota composition for each pig as described in the next section.

### Microbiota DNA preparation and sequencing

Since it has been shown that the microbiota composition of feces is similar to that of the large intestine [21], fecal samples were collected and analyzed to approximate the gut microbiota composition based on sequencing data of the ribosomal 16S DNA gene. Details of the microbial DNA preparation and sequencing are in Déru et al. [9], and briefly summarized in the next paragraph. After sequencing of the V3–V4 region of the 16S rRNA, high quality reads were filtered and trimmed using the DADA2 package in the R software [22]. Chimera were removed and no further clustering was applied, so in this study OTU were equivalent to amplicon sequence variants (ASV). Subsequently, the final OTU abundance table was obtained, followed by taxonomic annotation using the *assignTaxonomy* function of DADA2 with the Silva Dataset v132 [23]. The obtained file was rarefied to 10,000 counts per sample using the Phyloseq package [24] and had information on 1564 pigs. In total, 14,366 OTU were retained in the abundance tables for 812 pigs fed the CO diet and 752 pigs fed the HF diet. Sequence information obtained in the current study was deposited in the Short-Read Archive with accession number PRJNA741111.

### Genotyping

In total, 1691 animals were genotyped with the 70K single nucleotide polymorphism (SNP) GeneSeek GGP Porcine HD chip. The following quality control (QC) criteria were applied: a call rate per individual (the percentage of genotypes present per individual) of 95%, and a SNP call rate (the percentage of genotypes by SNP) of 95%. SNPs with a minor allele frequency lower than 5%, or that showed a significant deviation from Hardy–Weinberg equilibrium (*P* < 0.000001), and those on the sex chromosomes or not mapped were deleted. This QC was performed with the PLINK 1.09 software [25]. After QC, 1687 animals and 48,919 SNPs were available for further analyses.

### Statistical analyses

Only animals with microbiota and genomic data, and at least one phenotype, were kept for subsequent analyses, which left 1526 animals with 791 animals fed the CO diet and 735 fed the HF diet.

#### Microbiota and genomic covariance matrices

To account for the microbiota and genetics effects in the linear mixed models, a microbial covariance matrix based on microbiota data and a genomic relationship matrix were constructed.

The microbial covariance matrix was obtained under three editing conditions: without OTU filtering (14,366 OTU), by filtering the OTU present in more than five samples and with an average abundance higher than 0.001% (2399 OTU) or higher than 0.01% (803 OTU). The microbial covariance matrix (${\mathbf{M}}$) was computed for each diet separately and for the two diets jointly, and was obtained as follows:

$${\mathbf{M}} = \frac{{{\mathbf{SS}}^{{\text{T}}} }}{{\text{n}}},$$

where ${\mathbf{S}}$ is a matrix of dimension $p \times n$ with $p$ the number of animals and $n$ the number of OTU that depends on the filtering scenario, and is constructed from the elements $s_{jk}$, i.e. the log-transformed and standardized count for animal $j$ and OTU $k$ (plus 1.00) [11].

The genomic relationship ${\mathbf{G}}$ was computed for each diet separately and for the two diets jointly, and was constructed according to VanRaden’s first method [26] with the AGHmatrix package in R [27] from the ${\mathbf{Z}}$ matrix of genotypes coded 0, 1 and 2.

#### Models

We performed a preliminary analysis by ignoring the additive genomic and microbiota effects to determine the fixed and random effects to be included in subsequent analyses using a linear mixed model implemented with the “lme4” and “lmerTest” R packages [28, 29]. Only the effects that were significant at a threshold of 5% were retained. To estimate the contributions of host genetics and microbiota to the phenotypic variation of FE and DE traits, and the GEBV and EMV, we used three Bayesian linear regression models fitted with the BGLR package in R [30]: model Gen that included a genomic effect, model Micro that included a microbiota effect, and model Micro+Gen with both effects. Each model was fitted separately for the CO and HF diets and all were univariate linear mixed models.

The model that included only genomic information was:

model Gen $${\mathbf{y}} = {\mathbf{X}\user2{\upbeta}} + {\mathbf{Zu}} + {\mathbf{e}},$$

where ${\mathbf{y}}$ is the vector of phenotypes for a given trait; ${\mathbf{X}}$ is the incidence matrix relating observations to fixed effects; ${{\user2{\upbeta}}}$ is the vector of fixed effects depending on the trait considered, i.e. pen within batch and DFI for DC, pen within batch and weight at the end of the post-weaning phase for ADG and FCR, and pen within batch and weight at the end of the test for DFI; ${\mathbf{Z}}$ is the incidence matrix for the genetic effects; ${\mathbf{u}}\sim N\left( {{\mathbf{0}},{ }{\mathbf{G}}{\upsigma }_{{\text{u}}}^{2} } \right)$ is the vector of additive genetic random effects for the trait considered, with ${\mathbf{G}}$ the genomic relationship matrix and ${\upsigma }_{{\text{u}}}^{2}$ the additive genetic variance; and ${\mathbf{e}}\sim N\left( {{\mathbf{0}},{ }{\mathbf{I}}{\upsigma }_{{\text{e}}}^{2} } \right)$ is the vector of residual random effects, with ${\mathbf{I}}$ the incidence matrix, and ${\upsigma }_{{\text{e}}}^{2}$ the residual variance.

The model that included only microbiota information was:

model Micro $${\mathbf{y}} = {\mathbf{X}\user2{\upbeta}} + {\mathbf{Wm}} + {\mathbf{e}},$$

where ${\mathbf{X}}$, ${\user2{\upbeta}}$ and ${\mathbf{e}}$ are defined as for model Gen; ${\mathbf{W}}$ is the incidence matrix for the microbiota effects; and ${\mathbf{m}}\sim N\left( {{\mathbf{0}}, {\mathbf{M}}{\upsigma }_{{\text{m}}}^{2} } \right)$ is the vector of microbiota effects for the trait considered, with ${\mathbf{M}}$ the microbial covariance matrix, and ${\upsigma }_{{\text{m}}}^{2}$ the microbial variance.

The third model that jointly fitted genomic and microbiota information, with no covariance between the two random variables, was:

model Micro+Gen ${\mathbf{y}} = {\mathbf{X}\user2{\upbeta}} + {\mathbf{Zu}} + {\mathbf{Wm}} + {\mathbf{e}},$

where the effects are the same as in models (Gen) and (Micro), but jointly estimated.

A Bayesian reproducing kernel Hilbert space (RKHS) model was used. For all the models, the residual, microbiota, and genetic variances were assigned scaled-inverse Chi-square densities as prior density, with hyperparameters of 5 degrees of freedom and a scale parameter based on the sample variance of the phenotypes, as proposed by default in the BGLR package [30]. A Gaussian prior with mean zero and variance equal to 10^10^ was assigned to the fixed effects.

A single chain of 120,000 iterations was run for all the models, with 20,000 rounds discarded as burn-in and a thinning of 20. After discarding the burn-in, inferences on all the parameters were obtained from the mean of the respective posterior distributions. The results are presented as the means of the posterior distribution of the parameters of interest with their respective posterior standard deviation (SD). These standard deviations were then used to construct 95% highest density intervals (HDI) (means of the posterior distribution ± 1.96 * SD) to test differences between estimates.

For each trait, the three models were compared using the Bayesian information criterion (BIC), calculated as ${\text{BIC}} = - 2{\text{*Log}}\left( {\text{L}} \right) + {\text{k*Log}}\left( {\text{N}} \right)$, with ${\text{L}}$ being the log-likelihood evaluated at the posterior mean, the number of parameters estimated in the model and the sample size. The model with the lowest BIC was considered as the best.

#### Heritability and microbiability estimates

Heritability was computed as ${\text{h}}^{2} = \frac{{{\upsigma }^{2}_{{\text{u}}} }}{{{\upsigma }^{2}_{{\text{u}}} + {\upsigma }^{2}_{{\text{e}}} }}$ in the model Gen and as ${\text{h}}^{2} = \frac{{{\upsigma }^{2}_{{\text{u}}} }}{{\left( {{\upsigma }^{2}_{{\text{u}}} + {\upsigma }^{2}_{{\text{m}}} + {\upsigma }^{2}_{{\text{e}}} } \right)}}$ in the model Micro+Gen. Microbiability was computed as ${\text{ m}}^{2} = \frac{{{\upsigma }^{2}_{{\text{m}}} }}{{\left( {{\upsigma }^{2}_{{\text{m}}} { } + {\upsigma }^{2}_{{\text{e}}} } \right)}}$ in the model Micro and as ${\text{m}}^{2} = \frac{{{\upsigma }^{2}_{{\text{m}}} }}{{{\upsigma }^{2}_{{\text{m}}} + {\upsigma }^{2}_{{\text{u}}} + {\upsigma }^{2}_{{\text{e}}} }}$ in the model Micro+Gen. In this study, we arbitrarily categorized $h^{2}$ and $m^{2}$ estimates as low when they were lower than 0.20, as moderate when they ranged from 0.20 to 0.40, and as high when they were higher than 0.40.

#### Rank correlations

To evaluate the impact of adding microbiota information on GEBV, GEBV reranking was quantified using Spearman correlations between the Gen and Micro+Gen models. Similarly, EMV reranking was computed between the Micro and Micro+Gen models. Their 95% confidence intervals (CI) were determined for each rank correlation using a bootstrap approach implemented by the *spearman.ci* function in R, with 1000 replicates [19].

#### Genomic- and microbiota-by-diet interactions

Since BGLR does not allow multivariate implementations, the magnitude of the genomic-by-diet (G × D) and microbiota-by-diet deviations (M × D) for DE, FE and growth traits were estimated following Lopez-Cruz et al. [31]. The principle was to decompose variances into genetic variance (in the model G × D) and microbiota variance (in the model M × D) components estimated based on the two diets jointly (main effects) and the diet-specific components to compute an interaction term [31]. For this analysis, a single chain of 120,000 iterations was run for all models, with 20,000 rounds discarded as burn-in and a thinning of 20. Inferences on all parameters were obtained from the mean of the respective posterior distributions after discarding the burn-in. An R script for the computation of the G × D interaction for ADG is provided in Additional file 2: Script S1.

A univariate linear mixed model was applied to quantify the G × D interaction:

model G × D $$\left[ {\begin{array}{*{20}c} {{\mathbf{y}}_{{{\mathbf{CO}}}} } \\ {{\mathbf{y}}_{{{\mathbf{HF}}}} } \\ \end{array} } \right] = {\mathbf{X}\user2{\upbeta}} + { }\left[ {\begin{array}{*{20}c} {{\mathbf{Z}}_{{{\mathbf{CO}}}} } \\ {{\mathbf{Z}}_{{{\mathbf{HF}}}} } \\ \end{array} } \right]{\mathbf{b}}_{{\mathbf{0}}} + { }\left[ {\begin{array}{*{20}c} {{\mathbf{Z}}_{{{\mathbf{CO}}}} } & {\mathbf{0}} \\ {\mathbf{0}} & {{\mathbf{Z}}_{{{\mathbf{HF}}}} } \\ \end{array} } \right]\left[ {\begin{array}{*{20}c} {{\mathbf{b}}_{{{\mathbf{CO}}}} } \\ {{\mathbf{b}}_{{{\mathbf{HF}}}} } \\ \end{array} } \right] + { }\left[ {\begin{array}{*{20}c} {{\mathbf{e}}_{{{\mathbf{CO}}}} } \\ {{\mathbf{e}}_{{{\mathbf{HF}}}} } \\ \end{array} } \right],$$

with ${\mathbf{b}}_{{\mathbf{0}}} \sim N({\mathbf{0}},{\mathbf{I}}\sigma_{b0}^{2}$) and ${\mathbf{b}}_{{\mathbf{j}}} \sim N({\mathbf{0}},{\mathbf{I}}\sigma_{bj}^{2}$) being the main SNP effects and the specific SNP effects in each environment, respectively. ${\mathbf{Z}}_{{\mathbf{i}}}$ is the block of the full ${\mathbf{Z}}$ matrix corresponding to the animals raised in diet $i$ $\left( {I \in \left\{ {CO,HF} \right\}} \right)$. The model was implemented as follows:

model G × D $${\mathbf{y}} = {\mathbf{X}\user2{\upbeta}} + {\mathbf{u}}_{{\mathbf{0}}} + {\mathbf{u}}_{{\mathbf{1}}} + {\mathbf{e}},$$

where ${\mathbf{u}}_{{\mathbf{0}}} \sim N\left( {{\mathbf{0}},{\mathbf{G}}\upsigma _{{{\text{u}}0}}^{2} } \right)$ is the animal main genetic effects, and $${\mathbf{u}}_{{\mathbf{1}}} = \left[ {\begin{array}{*{20}c} {{\mathbf{u}}_{{{\mathbf{CO}}}} } \\ {{\mathbf{u}}_{{{\mathbf{HF}}}} } \\ \end{array} } \right]\sim N\left( {{\mathbf{0}},{\mathbf{G}}_{{\mathbf{1}}} } \right)$$ the environment specific genetic effects, with $${\mathbf{G}}_{{\mathbf{1}}} = {{\left[ {\begin{array}{*{20}c} {{\upsigma }_{{{\text{uCO}}}}^{2} {\mathbf{Z}}_{{{\mathbf{CO}}}} {{\mathbf{Z}}_{\mathbf{CO}}}^{\prime}} & 0 \\ 0 & {{\upsigma }_{{{\text{uHF}}}}^{2} {\mathbf{Z}}_{{{\mathbf{HF}}}} {{\mathbf{Z}}_{\mathbf{HF}}}^{\prime } } \\ \end{array} } \right]} \mathord{\left/ {\vphantom {{\left[ {\begin{array}{*{20}c} {{\upsigma }_{{{\text{uCO}}}}^{2} {\mathbf{Z}}_{{{\mathbf{CO}}}} {\mathbf{Z}}_{{{\mathbf{CO}}}}^{\prime } } & 0 \\ 0 & {{\upsigma }_{{{\text{uHF}}}}^{2} {\mathbf{Z}}_{{{\mathbf{HF}}}} {{\mathbf{Z}}_{\mathbf{HF}}}^{\prime}} \\ \end{array} } \right]} {\text{d}}}} \right. \kern-\nulldelimiterspace} {\text{d}}},$$ where ${\text{d}}$ is the number of SNPs.

The genetic-by-diet interaction was calculated as follows:

$${\text{r}}_{{\text{g}}} = \frac{{{\upsigma }^{2}_{{{\text{u}}0}} }}{{\sqrt {\left( {{\upsigma }^{2}_{{{\text{uCO}}}} + {\upsigma }^{2}_{{{\text{u}}0}} } \right)\left( {{\upsigma }^{2}_{{{\text{uHF}}}} + {\upsigma }^{2}_{{{\text{u}}0}} } \right)} }}.$$

Similarly, a univariate linear mixed model was applied to quantify the M × D interaction as:

model M × D $${\mathbf{y}} = {\mathbf{X}\user2{\upbeta}} + {\mathbf{m}}_{0} + {\mathbf{m}}_{1} + {\mathbf{e}},$$

where ${\mathbf{m}}_{0} \sim N \left( {{\mathbf{0}},{\mathbf{M}}{\upsigma }_{{{\text{m}}0}}^{2} } \right)$ is the vector of the animal main microbiota effects, and ${\mathbf{m}}_{1} \sim N\left( {{\mathbf{0}},{\mathbf{M}}_{1} } \right)$ the vector of the environment-specific microbiota effects, with $${\mathbf{M}}_{1} = {{\left[ {\begin{array}{*{20}c} {{\upsigma }_{{{\text{mCO}}}}^{2} {\mathbf{W}}_{{{\mathbf{CO}}}} {{\mathbf{W}}_{\mathbf{CO}}}^{\prime } } & 0 \\ 0 & {{\upsigma }_{{{\text{mHF}}}}^{2} {\mathbf{W}}_{{{\mathbf{HF}}}} {{\mathbf{W}}_{\mathbf{HF}}}^{\prime } } \\ \end{array} } \right]} \mathord{\left/ {\vphantom {{\left[ {\begin{array}{*{20}c} {{\upsigma }_{{{\text{mCO}}}}^{2} {\mathbf{W}}_{{{\mathbf{CO}}}} {\mathbf{W}}_{{{\mathbf{CO}}}}^{\prime } } & 0 \\ 0 & {{\upsigma }_{{{\text{mHF}}}}^{2} {\mathbf{W}}_{{{\mathbf{HF}}}} {\mathbf{W}}_{{{\mathbf{HF}}}}^{\prime } } \\ \end{array} } \right]} {\text{n}}}} \right. \kern-\nulldelimiterspace} {\text{n}}},$$ with ${\text{n}}$ the number of OTU.

The microbiota-by-diet interaction was calculated as follows:

$${\text{r}}_{{\text{m}}} = \frac{{{\upsigma }^{2}_{{{\text{m}}0}} }}{{\sqrt {\left( {{\upsigma }^{2}_{{{\text{mCO}}}} + {\upsigma }^{2}_{{{\text{m}}0}} } \right)\left( {{\upsigma }^{2}_{{{\text{mHF}}}} + {\upsigma }^{2}_{{{\text{m}}0}} } \right)} }}.$$

Mulder and Bijma [32] suggested that, when the genetic correlation between the principal and alternative environments was lower than 0.80, breeding schemes should be re-designed to account for outcomes in the alternative environment. Following this reference value, we considered that correlations lower than 0.80 were indicative of G × D or M × D interactions that should be accounted for in practical applications.

## Results

### Microbiabilities estimated for the three OTU filtering scenarios

The $m^{2}$ estimates obtained for the three OTU filtering scenarios (i.e. leaving 14,366, 2399, and 803 OTU) with model Micro are in Table 1 and the corresponding variance components for the random effects are in Additional file 1: Table S2.

**Table 1:** Microbiabilities estimated for three OTU filtering scenarios with Model Micro, i.e. without filtering (14,366 OTU), by filtering the OTU present in more than five samples and with an average abundance higher than 0.001% (2399 OTU), or higher than 0.01% (803 OTU)

	CO diet			HF diet		
	14,366 OTU	2399 OTU	803 OTU	14,366 OTU	2399 OTU	803 OTU
Feed efficiency and growth traits						
FCR	0.27 ± 0.08	0.20 ± 0.05	0.17 ± 0.04	0.14 ± 0.05	0.17 ± 0.05	0.14 ± 0.04
DFI	0.35 ± 0.06	0.35 ± 0.06	0.25 ± 0.05	0.41 ± 0.09	0.35 ± 0.06	0.29 ± 0.05
ADG	0.15 ± 0.05	0.20 ± 0.06	0.18 ± 0.05	0.30 ± 0.09	0.27 ± 0.07	0.21 ± 0.05
RFI	0.29 ± 0.08	0.22 ± 0.05	0.18 ± 0.04	0.28 ± 0.04	0.28 ± 0.07	0.18 ± 0.04
Digestive efficiency traits						
DC of energy	0.57 ± 0.07	0.48 ± 0.06	0.33 ± 0.05	0.74 ± 0.05	0.67 ± 0.06	0.50 ± 0.06
DC of organic matter	0.60 ± 0.07	0.47 ± 0.06	0.33 ± 0.05	0.75 ± 0.05	0.68 ± 0.06	0.49 ± 0.06
DC of nitrogen	0.73 ± 0.06	0.54 ± 0.02	0.49 ± 0.05	0.75 ± 0.05	0.67 ± 0.03	0.51 ± 0.06

CO diet: growing pigs fed a conventional diet; HF diet: growing pigs fed a high fiber diet

Microbiability values are the posterior means of the a posteriori distribution with their respective posterior standard deviations

*FCR* feed conversion ratio, *DFI* daily feed intake, *ADG* average daily gain, *RFI* residual feed intake, *DC* digestibility coefficient

Microbiability estimates were moderate for all scenarios and both diets for ADG (from 0.15 ± 0.05 to 0.30 ± 0.08), DFI (from 0.25 ± 0.05 to 0.41 ± 0.09), and FE (from 0.14 ± 0.04 to 0.29 ± 0.08), and those obtained within diet did not differ significantly between the OTU filtering scenarios. Thus, regardless of the filtering criteria applied, the microbiota had a similar effect on these traits.

For the DC, $m^{2}$ estimates decreased as the OTU filtering level increased, with posterior means ranging from 0.57 ± 0.03 to 0.75 ± 0.02 in the 14,366 OTU scenario, from 0.47 ± 0.02 to 0.68 ± 0.06 in the 2399 OTU scenario, and from 0.33 ± 0.02 to 0.51 ± 0.03 in the 803 OTU scenario. Within diet, the differences in $m^{2}$ estimates were significant between the two extreme models for the three DC, ranging from − 0.27 to − 0.24 points between the microbial covariance matrix constructed with 14,366 OTU and 803 OTU. An increase in the residual variance explained this decrease as the OTU filtering level increased and see Additional file 1: Table S2.

The microbial covariance matrix obtained with an intermediate number of OTU (2399) was used in the following analyses. The choice was based on the fact that this level of filtering provided intermediate $m^{2}$ estimates that did not differ from those obtained with the two extreme filtering levels. This filtering was similar to that proposed by Verschuren et al. [15] to estimate $m^{2}$ for DE traits, which allows a fair comparison between the two studies.

### Proportion of phenotypic variance explained by microbiota and host genetics within each diet

#### Comparison of the Bayesian information criterion between the three models

The BIC estimates obtained for the models with microbiota and genetic effects fitted separately and jointly are in Table 2. The model that best explained all FE and growth traits data was the Micro+Gen model, regardless of the diet, except for ADG for pigs fed the CO diet and FCR for pigs fed the HF diet. For these two traits, the best models were the Gen and Micro models, respectively. With the CO diet, the Micro model ranked second for all FE and growth traits, whereas with the HF diet, the Gen model ranked second for all FE and growth traits except for RFI. However, differences in BIC estimates between the Micro and Gen models were minimal in all cases, regardless of the diet. For the three DC and within each diet, the best model was Micro+Gen (BIC ranging from 2535 to 2779 for pigs fed the CO diet and from 2233 to 2373 for pigs fed the HF diet), followed by Micro (BIC ranging from 2547 to 2832 for pigs fed the CO diet and from 2272 to 2427 for pigs fed the HF diet).

**Table 2:** Bayesian information criterion (BIC) estimated for the models with the microbiota (Micro), and genetic effects (Gen) separately and jointly (Micro+Gen) for feed and digestive efficiency traits of growing pigs fed a conventional (CO) diet or a high fiber diet (HF) diet

	CO diet			HF diet		
	Micro	Gen	Micro+Gen	Micro	Gen	Micro+Gen
Feed efficiency and growth traits						
FCR	6570^a^	6574^b^	6559^c^	6282^c^	6283^a^	6293^b^
DFI	10,417^a^	10,423^b^	10,345^c^	9761^b^	9759^a^	9684^c^
ADG	9099^a^	9093^c^	9109^b^	8473^b^	8457^a^	8423^c^
RFI	9275^a^	9286^b^	9273^c^	8976^a^	8987^b^	8957^c^
Digestive efficiency traits						
DC of energy	2667^a^	2788^b^	2660^c^	2383^a^	2629^b^	2346^c^
DC of organic matter	2547^a^	2673^b^	2535^c^	2272^a^	2533^b^	2233^c^
DC of nitrogen	2832^a^	3060^b^	2779^c^	2427^a^	2674^b^	2373^c^

*FCR* feed conversion ratio, *DFI* daily feed intake, *ADG* average daily gain, *RFI* residual feed intake, *DC* digestibility coefficient

^a^Intermediate model (intermediate BIC)

^b^Worst model (highest BIC)

^c^Best model (lowest BIC)

#### Heritability and microbiability estimates

Heritability and $m^{2}$ estimated for the three models are in Table 3 and variances of the random effects for each model are in Additional file 1: Table S3.

**Table 3:** Posterior means of the phenotypic variance and their respective posterior standard deviations explained by the microbiota ($m^{2}$) and host genetics ($h^{2}$), separately, and jointly ($m^{2} + h^{2}$) using a Bayesian approach for feed and digestive efficiency traits of growing pigs fed a conventional (CO) diet or a high fiber (HF) diet

	CO diet				HF diet			
	Micro	Gen	Micro+Gen		Micro	Gen	Micro+Gen	
	$m^{2}$	$h^{2}$	$m^{2}$	${\text{h}}^{2}$	${\text{m}}^{2}$	$h^{2}$	$m^{2}$	$h^{2}$
Feed efficiency and growth traits								
FCR	0.20 ± 0.05	0.37 ± 0.07	0.14 ± 0.05	0.27 ± 0.05	0.17 ± 0.05	0.30 ± 0.07	0.14 ± 0.05	0.23 ± 0.05
DFI	0.35 ± 0.06	0.41 ± 0.07	0.42 ± 0.06	0.44 ± 0.04	0.35 ± 0.06	0.44 ± 0.07	0.28 ± 0.06	0.30 ± 0.04
ADG	0.20 ± 0.06	0.26 ± 0.06	0.17 ± 0.04	0.22 ± 0.05	0.27 ± 0.07	0.38 ± 0.07	0.20 ± 0.06	0.29 ± 0.04
RFI	0.22 ± 0.05	0.32 ± 0.07	0.18 ± 0.05	0.24 ± 0.05	0.28 ± 0.06	0.32 ± 0.07	0.33 ± 0.09	0.32 ± 0.09
Digestive efficiency traits								
DC of energy	0.44 ± 0.06	0.25 ± 0.05	0.39 ± 0.06	0.22 ± 0.04	0.67 ± 0.06	0.32 ± 0.06	0.59 ± 0.06	0.25 ± 0.04
DC of organic matter	0.44 ± 0.06	0.25 ± 0.06	0.40 ± 0.06	0.22 ± 0.04	0.68 ± 0.06	0.3 ± 0.08	0. 61 ± 0.06	0.24 ± 0.04
DC of nitrogen	0.60 ± 0.05	0.26 ± 0.07	0.55 ± 0.05	0.24 ± 0.04	0.67 ± 0.06	0.32 ± 0.08	0.61 ± 0.06	0.25 ± 0.04

$m^{2} = \sigma_{m}^{2} /\sigma_{p}^{2}$ as defined by Difford et al. [10]

*FCR* feed conversion ratio, *DFI* daily feed intake, *ADG* average daily gain, *RFI* residual feed intake, *DC* digestibility coefficient

For the FE, DFI and growth traits with model Micro, $m^{2}$ estimates were moderate for pigs fed the CO diet (from 0.20 ± 0.05 to 0.35 ± 0.06) and low to moderate for pigs fed the HF diet (from 0.17 ± 0.05 to 0.35 ± 0.06). Microbiability estimates did not differ significantly between diets for these traits. For the DE traits, $m^{2}$ estimates were high, i.e. from 0.44 ± 0.06 to 0.60 ± 0.05 and from 0.67 ± 0.06 to 0.68 ± 0.06) for pigs fed the CO and HF diets, respectively. For both DC of energy and organic matter, $m^{2}$ estimates were significantly higher for pigs fed the HF diet than for those fed the CO diet. This difference is due to the lower residual variance and higher microbiota variance for pigs fed the HF than those fed the CO diet. Microbiability estimates did not significantly differ between diets for the DC of nitrogen.

For DFI, RFI, FCR and ADG with model Gen, $h^{2}$ estimates were moderate to high, i.e. from 0.26 ± 0.06 to 0.41 ± 0.07 and from 0.30 ± 0.07 to 0.44 ± 0.07 for pigs fed the CO and HF diets, respectively. For these traits, the $h^{2}$ estimates obtained with the Gen model tended to be higher than the $m^{2}$ estimates obtained with the Micro model (+ 6 to 10% and + 4 to 13% for pigs fed the CO and HF diets, respectively). However, based on the 95% highest density intervals, these differences were not significant between the two models. For the DE traits, the $h^{2}$ estimates were moderate, i.e. from 0.25 ± 0.06 to 0.26 ± 0.07) and from 0.31 ± 0.08 to 0.32 ± 0.08 for pigs fed the CO and HF diets, respectively. Unlike the FE, DFI and growth traits, the $m^{2}$ estimates obtained with the Micro model were significantly higher than the $h^{2}$ estimates obtained with the Gen model for most DE traits (from + 19 to 34% and from + 35 to 37% for pigs fed the CO and HF diets, respectively), except for DC of energy and organic matter for pigs fed the CO diet, with higher but not significantly different estimates.

For all traits, the $m^{2}$ and $h^{2}$ estimates obtained with the Micro+Gen model were not significantly different from those obtained with the Micro and Gen models, even if the values were systematically lower.

### Rank correlations

#### Genomic estimated breeding values

Rank correlations of GEBV and their respective 95% CI computed with the Gen and the Micro+Gen models are in Table 4. Rank correlations were close to 1 for FE, DFI and growth traits, i.e. from 0.97 to 0.99 and from 0.95 to 0.99 for pigs fed the CO and HF diets, respectively. Rank correlations were lower for the DE traits, i.e. from 0.83 to 0.89 and from 0.78 to 0.84 for pigs fed the CO and HF diets, respectively. Thus, for the DE traits, including a microbiota effect had an impact on the ranking of the individuals’ GEBV.

**Table 4:** Rank correlations of genomic estimated breeding values (GEBV) and their respective 95% confidence intervals (CI) between the Gen and Micro+Gen models for feed and digestive efficiency traits recorded for pigs fed the conventional diet and the high fiber diet

Item	CO diet			HF diet		
	Spearman correlation	95% CI		Spearman correlation	95% CI	
		Lower	Upper		Lower	Upper
Feed efficiency and growth traits						
GEBV FCR	0.98	0.97	0.98	0.99	0.99	1.00
GEBV DFI	0.97	0.96	0.97	0.95	0.94	0.96
GEBV ADG	0.99	0.99	0.99	0.98	0.98	0.98
GEBV RFI	0.97	0.97	0.98	0.97	0.96	0.97
Digestive efficiency traits						
GEBV DC of energy	0.89	0.87	0.91	0.82	0.79	0.84
GEBV DC of organic matter	0.89	0.87	0.91	0.78	0.75	0.81
GEBV DC of nitrogen	0.83	0.80	0.85	0.84	0.81	0.86

*FCR* feed conversion ratio, *DFI* daily feed intake, *ADG* average daily gain, *RFI* residual feed intake, *DC* digestibility coefficient

#### Estimated microbiota values

Rank correlations for EMV are in Additional file 1: Table S4 and were close to 1 for all traits and each diet (0.96 to 0.99). Thus, no re-ranking of individuals according to their EMV is expected when a genetic effect is included.

### Genomic- and microbiota-by-feed interactions

Genomic and microbial correlations for traits between pigs fed a CO diet and those fed a HF diet are in Table 5. Genomic correlations estimates were high for the FE, DFI and growth traits (from 0.68 ± 0.09 to 0.81 ± 0.05), and moderate for the DE traits (from 0.35 ± 0.08 to 0.42 ± 0.10). Microbial correlations were moderate for the FE, DFI and growth traits (from 0.23 ± 0.09 to 0.41 ± 0.12), and moderate to high for the DE traits (from 0.46 ± 0.09 to 0.55 ± 0.09).

**Table 5:** Posterior means of genomic and microbial correlations and their respective standard deviations between pigs fed a conventional diet and a high fiber diet

	Genomic correlation	Microbial correlation
Feed efficiency and growth traits		
FCR	0.72 (0.06)	0.35 (0.10)
DFI	0.76 (0.05)	0.41 (0.12)
ADG	0.81 (0.05)	0.40 (0.13)
RFI	0.68 (0.09)	0.23 (0.09)
Digestive efficiency traits		
DC of energy	0.35 (0.08)	0.46 (0.09)
DC of organic matter	0.36 (0.08)	0.46 (0.09)
DC of nitrogen	0.42 (0.10)	0.55 (0.09)

*FCR* feed conversion ratio, *DFI* daily feed intake, *ADG* average daily gain, *RFI* residual feed intake, *DC* digestibility coefficient

## Discussion

Our results support the hypothesis that the fecal microbiota explains a substantial part of the phenotypic variability of FE and DE traits in growing pigs. When analyzed jointly, $m^{2}$ were lower or similar to $h^{2}$ for growth and FE traits, whereas $m^{2}$ were up to twice the $h^{2}$ for DE traits. Thus, our findings suggest that $m^{2}$ explains a large proportion of the phenotypic variance for DE traits, especially for pigs fed the HF diet.

### Differences in microbiability between traits and between diets

The $m^{2}$ estimates obtained in this study were moderate for the growth, DFI and FE traits and high for the DE traits and are consistent with those found in the literature. Camarinha-Silva et al. [12] and Lu et al. [13] reported $m^{2}$ estimates of ~ 0.20 for FCR, DFI and growth traits (ADG). Aliakbari et al. [33] found $m^{2}$ estimates of the same order of magnitude for FE traits in divergent lines of Large White pigs selected on RFI, i.e. 0.11 ± 0.09 for RFI and 0.20 ± 0.11 for FCR. Conversely, in the previously cited study [33], estimates were lower and close to 0 for DFI (0.04 ± 0.03) and ADG (0.03 ± 0.03). Using data from Camarinha-Silva et al. [12], Weishaar et al. [14] estimated a higher $m^{2}$ for RFI (0.45 ± 0.15) than in our study. Based on fecal microbiota samples that were collected from 160 growing pigs from a three-way cross, Verschuren et al. [15] estimated high $m^{2}$ values for DC of dry matter and organic matter (0.59 ± 0.19 and 0.58 ± 0.19, respectively), and even higher values for DC of crude protein (0.93 ± 0.10). Our results confirmed these high $m^{2}$ values for DE traits on a larger dataset. Microbiota is closely related to the digestion process. Due to its fermentation activity, the gut microbiota enables the production of primary and secondary metabolites (such as short-chain fatty acids, vitamins, metabolites, etc.), which are then absorbed at the level of the intestinal barrier [6, 7]. This biological interaction between microbiota composition and digestion could explain the high $m^{2}$ estimates found for the DE traits. Microbiability values estimated for ADG, DFI and FE traits were similar and not significantly different between the two diets. Microbiability estimates for DC of energy and organic matter were significantly higher for pigs fed the HF diet than for those fed the CO diet (approximately 25% higher). These findings suggest that the composition of the gut microbiota is more critical for digesting feed with a high fiber content, which is consistent with the high fermentation activity in the large intestine of animals fed a HF diet. Indeed, when dietary fibers are present in the feed, many indigestible polysaccharides arrive intact in the large intestine where they undergo fermentation and are then degraded, in particular, in volatile fatty acids (VFA) (mainly propionate, acetate and butyrate) [7]. Subsequently, in pigs, 95% of the VFA are absorbed in the large intestine [34]. Thus, the higher fermentation activity of the gut microbiota in the presence of dietary fibers might explain the higher $m^{2}$ values observed for pigs fed the HF diet compared to those fed the CO diet.

Thus, our results confirm that the phenotypic variance of FE and growth traits is better explained by the host additive genetic effects than by the microbiota effects, as already reported in the literature [12, 33]. In contrast, the phenotypic variance of the DE traits was better explained by the microbiota effects than the host genetics effects, especially for pigs fed the HF diet.

### Partition of the variance between the microbial covariance matrix and the genomic relationship matrix

In this study, the $m^{2}$ and $h^{2}$ estimates obtained in the Micro+Gen model did not significantly differ from those obtained in the Gen and Micro models for all traits. In addition, the rank correlations of EMV between the Micro and Micro+Gen models were close to 1 for all traits. Similarly, the rank correlations of GEBV between the Gen and Micro+Gen models were close to 1 for the FE traits. To our knowledge, only the study of Aliakbari et al. [33] found non-significantly different $m^{2}$ and $h^{2}$ estimates between models that fit the effects jointly and separately. Our study did not suggest microbiota-host confounding for FE traits in pigs. We observed a slight reranking for the DE traits but to date no other study has reported $h^{2}$ and $m^{2}$ estimates for DE traits using a model that fits the microbiota and host genetics effects jointly. David and Ricard [35] showed that accounting for all the effects that could be partially confounded with a genetic additive effect is necessary for accurate EBV predictions. Thus, one hypothesis is that the addition of microbial information in the model allows a better estimation of the GEBV, which could be the case for the DE traits. However, taken together, our results do not allow us to conclude whether partial confounding between the microbiota and genomic effects is present or not in the models that fit the effects separately. In addition, the comparison of the models in our study was based on the BIC only, while the accuracy of predictions of GEBV and EMV should also be used since they can affect the reranking of GEBV and EMV. For carcass composition and meat quality traits, Khanal et al. [36] observed that the genomic variance was eroded when microbiota information was included in the model, which suggested a possible overlap between the microbiota and host genetics effects. To consider this potential confounding between microbiota and genetic effects, Khanal et al. [36] included a genetic-by-microbiota interaction term in the model, in order to separate the transmissible and non-transmissible components of the microbiota. Similarly, Weishaar et al. [14] and Christensen et al. [37] proposed formal decompositions of the microbiota contribution to trait variances into genetic and non-genetic components. These methods could better separate and explain the GEBV of traits that are influenced by both the microbiota and the host genetics.

The Micro+Gen model was the best in terms of goodness-of-fit for all traits except for ADG for pigs fed the CO diet and for FCR for pigs fed the HF diet. This result suggests that the microbiota and the host genetics provide complementary information to better explain the variances of FE and DE traits. To confirm this, the predictive ability of the Micro+Gen model to predict phenotypes should be estimated and compared with that of the Micro and Gen models. Using simulated phenotypes in cattle, Pérez-Enciso et al. [38] showed that including both microbiota and genome data in the model could increase the predictive accuracy by 50%. In addition, such comparisons could confirm if a model that fits jointly microbiota and genomic effects can help better predict digestive and feed efficiency phenotypes than models fitting each effect separately. Indeed, Maltecca et al. [39] suggested that microbiota can be used to predict phenotypes for growth traits in swine that are fed easy-to-digest diets. Camarinha-Silva et al. [12] showed that microbiota predictions tended to be more accurate than genomic predictions for ADG, FCR and DFI, although not significantly. For DC of dry matter, organic matter, crude protein and non-starch polysaccharide, Verschuren et al. [15] found that the accuracies of microbiota predictions were high (from 0.42 to 0.63).

### Methodological developments for the efficient use of microbiota data

#### Impact of OTU filtering

For the DE traits, the number of OTU used to construct the microbial covariance matrix, for both the CO and HF diets, significantly impacted the $m^{2}$ estimates. Therefore, the larger is the number of OTU considered, the larger is the proportion of variance of the DE traits explained by the microbiota. This finding is consistent with the high genetic correlations estimated between microbiota diversity indices and DE traits (from 0.88 ± 0.12 to 0.91 ± 0.13) as reported by Déru et al. [9]. Thus, many OTU are required to explain the phenotypic variation of DE traits, thus imposing strict editing rules eliminates relevant information that contributes to the variation of DE traits.

Conversely, the $m^{2}$ estimates were not significantly different between the three OTU filtering scenarios for ADG and FE traits. Thus, most of the phenotypic variation was captured in the scenario with the 803 most common OTU for these traits. Moreover, in the three filtering scenarios resulting in 14,366 to 803 OTU, the OTU with the lowest abundances and therefore the rarest ones were eliminated. One hypothesis could be that (some of) the rarest OTU are specific to some features of the digestion process that are not captured by the main OTU, which would explain why the $m^{2}$ estimates decrease when these are no longer taken into account to construct the microbial covariance matrix. In addition, the differences in timing of sample collection for DE traits, microbiota and FE traits could also have an impact on these observed differences. Samples of feces to determine microbiota and DE traits were collected at the same time and in the middle of the control period, while the FE and growth traits were measured throughout the control period. One hypothesis is that the microbiota data better reflected the composition of the gut microbiota for the DE traits than for the FE and ADG traits because for the former sampling of feces took place at the same time. Thus, this could explain why the $m^{2}$ estimates did not significantly differ between the three OTU filtering scenarios for the FE and ADG traits. Further investigation is necessary to determine if the $m^{2}$ estimates for the DE traits remain high when the fecal samples used to evaluate DE traits and microbiota composition are not collected at the same time.

In the literature, different criteria have been applied to edit microbiota data [10, 15, 40]. According to our study, OTU filtering criteria may have a different impact on $m^{2}$ estimates depending on the trait, i.e. a limited impact was observed for the FE traits whereas it was stronger for the DE traits. Nevertheless, further studies are needed to confirm our findings and to determine an optimal editing method for OTU that could be used and facilitate comparisons between studies, especially for traits linked to digestion.

#### Choices for the construction of the covariance microbial matrix

In the literature, $m^{2}$ estimates have been obtained with different procedures to build the covariance microbial matrix, such as the use of a Bray–Curtis dissimilarity matrix as relationship matrix [41], or of the variance–covariance from the log-transformed OTU [12, 15, 40, 41], as suggested by Ross et al. [11]. In our study we used log-transformed OTU. However, it should be noted that Ramayo-Caldas et al. [41] reported higher $m^{2}$ estimates using Bray–Curtis-based kernels with the RKHS model than the log-transformed covariance matrix of abundances, which confirms that the method used to construct the microbial covariance matrix has an impact on $m^{2}$ estimates.

#### Modeling the microbiota effect with alternative methods

Microbiality estimates may be affected by the underlying model used for the distribution of the microbial effects, or by the estimation framework. Numerous methods exist to estimate $m^{2}$, such as the Bayes C hypotheses of mixtures of distributions with different variances [38], MBLUP [18], and Bayesian regression [12, 15, 40, 41]. Pérez-Enciso et al. [38] compared $m^{2}$ estimates obtained with Bayes C and Bayesian RKHS. With the Bayes C method, they observed that the $m^{2}$ estimates can be biased, especially if $m^{2}$ is higher than 0.25, but for $m^{2}$ close to 0, they were well estimated. In contrast, the estimates obtained with the Bayesian RKHS methodology were slightly less biased and less sensitive to the number of causative OTU. However, estimates of variance fractions in the order of 0.10 were obtained even if the true variance was zero [38]. More studies are needed to compare different models and their impact on the estimation of $m^{2}$, to facilitate future comparisons of results between studies.

### Implications for selection

Digestive efficiency is known to be a trait of interest to improve feed efficiency, especially with the diversification of feed resources [5]. The existence of a moderate to high microbiability for traits of interest in pigs represents a new opportunity for breeding programs. Adding microbiota information in the model had no impact on rank correlations of GEBV for growth, DFI and FE traits but had an impact for the DE traits, especially for pigs fed the HF diet. This observation is consistent with the genetic × diet correlations estimated for DE traits in this study, which will help to better understand the interactions between host genetics and feed, provided that the main and diet-specific effects can be identified and properly estimated with this univariate computation. The advantage of this analysis is that the variance is separated in a common component between the two diets that corresponds to the covariance between diets, and in specific components due to the diets. However, these results need to be confirmed on a larger dataset. In addition, since the microbiability estimate was high for the DE traits, selection for microbiota genera that are favorably associated with digestive efficiency could also be considered and thus improve feed efficiency and growth performances in pigs as suggested in Déru et al. [9]. Additional work is needed to investigate these dynamics. Furthermore, in this work we used fecal samples to collect gut microbiota information, which is a non-invasive method, justifying its potential use for breeding purposes. Finally, to select DE traits, especially in a context of diversification of feed resources, collecting microbiota information to better estimate GEBV for these traits is probably relevant.

## Conclusions

In conclusion, the microbiota composition provides valuable information to better explain the phenotypic variation of DE traits, and to a lesser extent of FE traits, and thus to obtain a more accurate classification of breeders. Microbiota explained a large part of the phenotypic variance of DE traits with higher $m^{2}$ estimates for pigs fed a HF diet. However, microbiota explained a moderate part of the phenotypic variance of FE and growth traits, which was lower than that explained by the host genetics. Digestibility has already been highlighted as an attractive trait to be included in breeding schemes, since, in the future, pigs will be fed diets with a high fiber content [5]. Microbial composition explained a moderate to large proportion of the phenotypic variance, especially for the DE traits, which implies that the inclusion of microbiota in animal evaluations might accelerate the genetic gain for these traits.

## Supplementary Information

**Additional file 1: Table S1.** Composition (%) of the conventional (CO) and the high fiber (HF) diets. Description of the ingredient composition for pigs fed a conventional and a high-fiber diet. **Table S2.** Variance components for random effects included in the model with the microbial covariance matrix with 14,366 OTU (Scenario 1), 2399 OTU (Scenario 2) and 803 OTU (Scenario 3) using a Bayesian approach for feed and digestive efficiency traits in growing pigs fed a conventional (CO) diet or a high fiber (HF) diet, along with their posterior standard deviation. **Table S3.** Variance components for random effects included in the model with microbiota (model Micro), genetics (model Gen) and microbiota and genetics jointly (model Micro+Gen) using a Bayesian approach for feed and digestive efficiency traits in growing pigs fed a conventional (CO) diet or a high fiber (HF) diet, along with their posterior standard deviation. **Table S4.** Rank correlations of estimated microbiota values (EMV) between the model with only microbiota and the model with genetic and microbiota for feed and digestive efficiency traits records in the conventional diet and in the high fiber diet, along with their 95% confidence intervals (CI). Rank correlations of estimated microbiota values were compared between two models, with and without genomic information in two diets.

**Additional file 2: Script S1.** Script to implement the genetic-by-diet interaction model—example for ADG. Detailed script for the example of the G × D interaction for ADG.

## Data Availability

The data that support the findings of this study are available on request from the corresponding author. The data are not publicly available due to privacy or ethical restrictions. Only the sequences obtained for microbiota are available and submitted to the Short-Read Archive with accession number PRJNA741111 (https://www.ncbi.nlm.nih.gov/sra/PRJNA741111).

## References

[1] Patience JF, Rossoni-Serão MC, Gutiérrez NA (2015). A review of feed efficiency in swine: biology and application. J Anim Sci Biotechnol.

[2] Quiniou N, Noblet J (2012). Effect of the dietary net energy concentration on feed intake and performance of growing–finishing pigs housed individually. J Anim Sci.

[3] Déru V, Bouquet A, Hassenfratz C, Blanchet B, Carillier-Jacquin C, Gilbert H (2020). Impact of a high-fibre diet on genetic parameters of production traits in growing pigs. Animal.

[4] Sevillano CA, Nicolaiciuc CV, Molist F, Pijlman J, Bergsma R (2018). Effect of feeding cereals–alternative ingredients diets or corn–soybean meal diets on performance and carcass characteristics of growing–finishing gilts and boars. J Anim Sci.

[5] Déru V, Bouquet A, Labussière E, Ganier P, Blanchet B, Carillier-Jacquin C (2021). Genetics of digestive efficiency in growing pigs fed a conventional or a high-fibre diet. J Anim Breed Genet.

[6] Broom LJ, Kogut MH (2018). Gut immunity: its development and reasons and opportunities for modulation in monogastric production animals. Anim Health Res Rev.

[7] Gardiner GE, Metzler-Zebeli BU, Lawlor PG (2020). Impact of intestinal microbiota on growth and feed efficiency in pigs: a review. Microorganisms.

[8] Caprita A, Căpriţă R, Simulescu V, Drehe RM (2010). Dietary fiber: chemical and functional properties. J Agroaliment Process Technol.

[9] Déru V, Bouquet A, Zemb O, Blanchet B, De Almeida ML, Cauquil L, et al. Genetic relationships between efficiency traits and gut microbiota traits in growing pigs fed a conventional or a high fiber diet. 2021. 10.1101/2021.11.15.468583. 10.1093/jas/skac183 PMC9194801 35579995

[10] Difford GF, Plichta DR, Løvendahl P, Lassen J, Noel SJ, Højberg O (2018). Host genetics and the rumen microbiome jointly associate with methane emissions in dairy cows. PLoS Genet.

[11] Ross EM, Moate PJ, Marett L, Cocks BG, Hayes BJ (2013). Investigating the effect of two methane-mitigating diets on the rumen microbiome using massively parallel sequencing. J Dairy Sci.

[12] Camarinha-Silva A, Maushammer M, Wellmann R, Vital M, Preuss S, Bennewitz J (2017). Host genome influence on gut microbial composition and microbial prediction of complex traits in pigs. Genetics.

[13] Lu D, Tiezzi F, Schillebeeckx C, McNulty NP, Schwab C, Shull C (2018). Host contributes to longitudinal diversity of fecal microbiota in swine selected for lean growth. Microbiome.

[14] Weishaar R, Wellmann R, Camarinha-Silva A, Rodehutscord M, Bennewitz J (2020). Selecting the hologenome to breed for an improved feed efficiency in pigs—a novel selection index. J Anim Breed Genet.

[15] Verschuren LMG, Schokker D, Bergsma R, Jansman AJM, Molist F, Calus MPL (2020). Prediction of nutrient digestibility in grower-finisher pigs based on faecal microbiota composition. J Anim Breed Genet.

[16] Déru V, Bouquet A, Zemb O, Blanchet B, Carillier-Jacquin C, Gilbert H. Influence d’une alimentation avec une teneur accrue en fibres sur le microbiote intestinal du porc en croissance. In Proceedings of the 53^emes^ Journées de la Recherche Porcine: 1–4 February 2021; virtual meeting. https://www.youtube.com/watch?v=YDrLmDN7f1c. Accessed 6 Dec 2021.

[17] Zhao J, Bai Y, Tao S, Zhang G, Wang J, Liu L (2019). Fiber-rich foods affected gut bacterial community and short-chain fatty acids production in pig model. J Funct Foods.

[18] Difford G, Lassen J, Lovendahl P. Genes and microbes, the next step in dairy cattle breeding. In: Proceedings of the 67th annual meeting of the European federation of animal science: 29 August–3 September 2016; Belfast. 2016.

[19] R Core Team (2016). R: a language and environment for statistical computing.

[20] Labussière E, Ganier P, Conde JA, Janvier E, Van Milgen JJ. Development of a NIRS method to assess the digestive ability in growing pigs. In: Proceedings of the 67th annual meeting of the European federation of animal science: 29 August–3 September 2016; Belfast. 2016.

[21] Zhao W, Wang Y, Liu S, Huang J, Zhai Z, He C (2015). The dynamic distribution of porcine microbiota across different ages and gastrointestinal tract segments. PLoS One.

[22] Callahan BJ, McMurdie PJ, Rosen MJ, Han AW, Johnson AJA, Holmes SP (2016). DADA2: high-resolution sample inference from Illumina amplicon data. Nat Methods.

[23] Quast C, Pruesse E, Yilmaz P, Gerken J, Schweer T, Yarza P (2013). The SILVA ribosomal RNA gene database project: improved data processing and web-based tools. Nucleic Acids Res.

[24] McMurdie PJ, Holmes S (2013). phyloseq: an R package for reproducible interactive analysis and graphics of microbiome census data. PLoS One.

[25] Purcell S, Neale B, Todd-Brown K, Thomas L, Ferreira MAR, Bender D (2007). PLINK: a tool set for whole-genome association and population-based linkage analyses. Am J Hum Genet.

[26] VanRaden PM (2008). Efficient methods to compute genomic predictions. J Dairy Sci.

[27] Amadeu RR, Cellon C, Olmstead JW, Garcia AAF, Resende MFR, Muñoz PR (2016). AGHmatrix: R package to construct relationship matrices for autotetraploid and diploid species: a blueberry example. Plant Genome.

[28] Kuznetsova A, Brockhoff PB, Christensen RHB (2017). lmerTest package: tests in linear mixed effects models. J Stat Soft.

[29] Bates D, Mächler M, Bolker B, Walker S (2015). Fitting linear mixed-effects models using lme4. J Stat Soft.

[30] Pérez P, de los Campos G (2014). Genome-wide regression and prediction with the BGLR statistical package. Genetics.

[31] Lopez-Cruz M, Crossa J, Bonnett D, Dreisigacker S, Poland J, Jannink JL (2015). Increased prediction accuracy in wheat breeding trials using a marker × environment interaction genomic selection model. G3 (Bethesda).

[32] Mulder HA, Bijma P (2005). Effects of genotype × environment interaction on genetic gain in breeding programs. J Anim Sci.

[33] Aliakbari A, Zemb O, Cauquil L, Barilly C, Billon Y, Gilbert H (2022). Microbiability and microbiome-wide association analyses of feed efficiency and performance traits in pigs. Genet Sel Evol.

[34] Argenzio RA, Southworth M (1975). Sites of organic acid production and absorption in gastrointestinal tract of the pig. Am J Physiol.

[35] David I, Ricard A (2019). A unified model for inclusive inheritance inlivestock species. Genetics.

[36] Khanal P, Maltecca C, Schwab C, Fix J, Bergamaschi M, Tiezzi F (2020). Modeling host-microbiome interactions for the prediction of meat quality and carcass composition traits in swine. Genet Sel Evol.

[37] Christensen OF, Börner V, Varona L, Legarra A (2021). Genetic evaluation including intermediate omics features. Genetics.

[38] Pérez-Enciso M, Zingaretti LM, Ramayo-Caldas Y, de los Campos G (2021). Opportunities and limits of combining microbiome and genome data for complex trait prediction. Genet Sel Evol.

[39] Maltecca C, Lu D, Schillebeeckx C, McNulty NP, Schwab C, Shull C (2019). Predicting growth and carcass traits in swine using microbiome data and machine learning algorithms. Sci Rep.

[40] Khanal P, Maltecca C, Schwab C, Fix J, Tiezzi F (2021). Microbiability of meat quality and carcass composition traits in swine. J Anim Breed Genet.

[41] Ramayo-Caldas Y, Zingaretti L, Popova M, Estellé J, Bernard A, Pons N (2020). Identification of rumen microbial biomarkers linked to methane emission in Holstein dairy cows. J Anim Breed Genet.

