# Supplementary material for: Gut microbiota and host genetics contribute to the phenotypic variation of digestive and feed efficiency traits in growing pigs fed a conventional and a high fiber diet
Source: Genet Sel Evol. 2022 Jul 27;54:55. doi: 10.1186/s12711-022-00742-6 (PMC9327178; doi:10.1186/s12711-022-00742-6)
Supplement: Supplementary file 1 — Additional file 1: Table S1. Composition (%) of the conventional (CO) and the high fiber (HF) diets. Description of the ingredient composition for pigs fed a conventional and a high-fiber diet. Table S2. Variance components for random effects included in the model with the microbial covariance matrix with 14,366 OTU (Scenario 1), 2399 OTU (Scenario 2) and 803 OTU (Scenario 3) using a Bayesian approach for feed and digestive efficiency traits in growing pigs fed a conventional (CO) diet or a high fiber (HF) diet, along with their posterior standard deviation. Table S3. Variance components for random effects included in the model with microbiota (model Micro), genetics (model Gen) and microbiota and genetics jointly (model Micro+Gen) using a Bayesian approach for feed and digestive efficiency traits in growing pigs fed a conventional (CO) diet or a high fiber (HF) diet, along with their posterior standard deviation. Table S4. Rank correlations of estimated microbiota values (EMV) between the model with only microbiota and the model with genetic and microbiota for feed and digestive efficiency traits records in the conventional diet and in the high fiber diet, along with their 95% confidence intervals (CI). Rank correlations of estimated microbiota values were compared between two models, with and without genomic information in two diets. [file 12711_2022_742_MOESM1_ESM.docx]

**Additional file 1**

**Table S1 Ingredient composition (%) of the conventional (CO) and the high fiber (HF) diets**

|  | Growing phase | |  | Finishing phase | |
| --- | --- | --- | --- | --- | --- |
| Ingredients (%) | CO diet | HF diet |  | CO diet | HF diet |
| Wheat | 38.29 | 38.00 |  | 42.57 | 39.30 |
| Corn | 25.00 | 0.00 |  | 25.00 | 0.00 |
| Barley | 15.00 | 16.87 |  | 15.00 | 17.60 |
| Rapeseed meal | 6.00 | 6.00 |  | 10.00 | 9.90 |
| Sunflower meal no shelled | 3.00 | 3.00 |  | 4.80 | 3.00 |
| Soybean meal, 48% CP | 10.4 | 5.40 |  | 2.50 | 0.00 |
| Wheat bran | 0.00 | 15.00 |  | 0.00 | 15.00 |
| Soybean hulls | 0.00 | 8.00 |  | 0.00 | 8.00 |
| Beet pulp | 0.00 | 5.00 |  | 0.00 | 5.00 |
| _L-_Lys HCL | 0.44 | 0.35 |  | 0.11 | 0.31 |
| _DL-_Met | 0.09 | 0.03 |  | 0.01 | 0.00 |
| _L-_Thr | 0.13 | 0.11 |  | 0.02 | 0.10 |
| Pure valine | 0.02 | 0.00 |  | 0.00 | 0.00 |
| Calcium carbonate | 1.40 | 1.12 |  | 0.12 | 1.01 |
| Dicalcium phosphate | 0.49 | 0.29 |  | 0.05 | 0.00 |
| NaCl | 0.40 | 0.40 |  | 0.40 | 0.40 |
| Vitamin and trace mineral mixture 0.5% | 0.40 | 0.40 |  | 0.40 | 0.40 |

**Table S2 Variance components for random effects included in the model with microbial relationship matrix with 14,366 OTU (Scenario 1), 2,399 OTU (Scenario 2) and 803 OTU (Scenario 3) using a Bayesian approach for feed and digestive efficiency traits in growing pigs fed a conventional (CO) diet or a high fiber (HF) diet, along with their posterior standard deviation**

|  | Scenario 1 – 14,366 OTUs | |  | Scenario 2 – 2,399 OTUs | |  | Scenario 3 – 803 OTUs | |
| --- | --- | --- | --- | --- | --- | --- | --- | --- |
| Traits | CO diet | HF diet |  | CO diet | HF diet |  | CO diet | HF diet |
| FCR, kg/kg |  |  |  |  |  |  |  |  |
| Microbiota variance | 58 (8) | 42 (6) |  | 44 (5) | 49 (7) |  | 28 (3) | 38 (5) |
| Residual variance | 157 (8) | 249 (9) |  | 173 (6) | 246 (8) |  | 233 (4) | 181 (5) |
| DFI, kg/day |  |  |  |  |  |  |  |  |
| Microbiota variance | 10673 (789) | 15346 (1568) |  | 11054 (1048) | 13150 (1230) |  | 8000 (789) | 11293 (1097) |
| Residual variance | 20149 (704) | 22197 (1443) |  | 20208 (840) | 24812 (1046) |  | 23411 (704) | 24812 (1046) |
| ADG, g/day |  |  |  |  |  |  |  |  |
| Microbiota variance | 851 (122) | 1825 (249) |  | 1161 (161) | 1671 (213) |  | 1036 (132) | 1321 (167) |
| Residual variance | 4651 (162) | 4298 (242) |  | 4446 (159) | 4562 (189) |  | 4656 (137) | 4972 (158) |
| RFI, g/day |  |  |  |  |  |  |  |  |
| Microbiota variance | 3703 (476) | 5265 (731) |  | 2826 (329) | 5380 (648) |  | 2300 (266) | 3470 (431) |
| Residual variance | 8996 (481) | 13638 (746) |  | 9960 (340) | 13813 (577) |  | 10571 (305) | 15366 (459) |
| DC of energy, % |  |  |  |  |  |  |  |  |
| Microbiota variance | 2.12 (0.14) | 3.10 (0.14) |  | 1.58 (0.12) | 2.84 (0.16) |  | 1.21 (0.10) | 1.76 (0.13) |
| Residual variance | 1.58 (0.11) | 1.10 (0.10) |  | 2.02 (0.09) | 1.38 (0.10) |  | 2.39 (0.08) | 1.83 (0.09) |
| DC of organic matter, % |  |  |  |  |  |  |  |  |
| Microbiota variance | 1.82 (0.11) | 2.62 (0.12) |  | 1.32 (0.11) | 2.40 (0.12) |  | 0.97 (0.08) | 2.40 (0.14) |
| Residual variance | 1.23 (0.09) | 0.87 (0.08) |  | 1.65 (0.08) | 1.12 (0.08) |  | 1.67 (0.07) | 1.12 (0.08) |
| DC of nitrogen, % |  |  |  |  |  |  |  |  |
| Microbiota variance | 4.04 (0.19) | 3.43 (0.15) |  | 3.22 (0.19) | 3.02 (0.17) |  | 2.58 (0.18) | 2.37 (0.18) |
| Residual variance | 1.50 (0.14) | 1.12 (0.10) |  | 2.08 (0.08) | 1.49 (0.11) |  | 2.66 (0.10) | 2.24 (0.10) |

FCR = feed conversion ratio; DFI = daily feed intake; ADG = average daily gain; RFI = residual feed intake; DC = digestibility coefficient

**Table S3 Variance components for random effects included in the model with microbiota (model Micro), genetics (model Gen) and microbiota and genetics jointly (model Micro+Gen) using a Bayesian approach for feed and digestive efficiency traits in growing pigs fed a conventional (CO) diet or a high fiber (HF) diet, along with their posterior standard deviation**

|  | Model Micro | |  | Model Gen | |  | Model Micro+Gen | |
| --- | --- | --- | --- | --- | --- | --- | --- | --- |
| Traits | CO diet | HF diet |  | CO diet | HF diet |  | CO diet | HF diet |
| FCR, kg/kg |  |  |  |  |  |  |  |  |
| Microbiota variance | 44 (5) | 49 (7) |  | - | - |  | 38 (11) | 43 (15) |
| Genetic variance | - | - |  | 81 (19) | 91 (25) |  | 69 (18) | 81 (23) |
| Residual variance | 173 (6) | 246 (8) |  | 139 (15) | 202 (22) |  | 113 (16) | 176 (23) |
| DFI, kg/day |  |  |  |  |  |  |  |  |
| Microbiota variance | 11054 (1048) | 13150 (1230) |  | - | - |  | 9015 (742) | 10779 (903) |
| Genetic variance | - | - |  | 12952 (2455) | 17160 (3280) |  | 9843 (2054) | 12451 (2740) |
| Residual variance | 20208 (840) | 24812 (1046) |  | 18463 (1938) | 21485 (2510) |  | 12386 (1835) | 14903 (2369) |
| ADG, g/day |  |  |  |  |  |  |  |  |
| Microbiota variance | 1161 (161) | 1671 (213) |  | - | - |  | 961 (318) | 1287 (381) |
| Genetic variance | - | - |  | 1432 (369) | 2377 (504) |  | 1315 (367) | 2201 (482) |
| Residual variance | 4446 (159) | 4562 (189) |  | 4033 (355) | 3771 (410) |  | 3369 (399) | 2790 (432) |
| RFI, g/day |  |  |  |  |  |  |  |  |
| Microbiota variance | 2826 (329) | 5380 (648) |  | - | - |  | 2333 (659) | 4923 (1490) |
| Genetic variance | - | - |  | 4219 (1069) | 6168 (1558) |  | 3327 (957) | 4726 (1407) |
| Residual variance | 9960 (340) | 13813 (577) |  | 8732 (921) | 13093 (1382) |  | 7212 (907) | 9720 (1559) |
| DC of energy, % |  |  |  |  |  |  |  |  |
| Microbiota variance | 1.58 (0.12) | 2.84 (0.16) |  | - | - |  | 1.46 (0.25) | 2.56 (0.34) |
| Genetic variance | - | - |  | 1.01 (0.24) | 1.63 (0.45) |  | 0.64 (0.15) | 0.61 (0.15) |
| Residual variance | 2.02 (0.09) | 1.38 (0.10) |  | 3.05 (0.26) | 3.38 (0.41) |  | 1.61 (0.21) | 1.14 (0.20) |
| DC of organic matter, % |  |  |  |  |  |  |  |  |
| Microbiota variance | 1.32 (0.11) | 2.40 (0.12) |  | - | - |  | 0.97 (0.08) | 2.40 (0.14) |
| Genetic variance | - | - |  | 0. 84 (0.20) | 1.29 (0.38) |  |  |  |
| Residual variance | 1.65 (0.08) | 1.12 (0.08) |  | 2.52 (0.22) | 2.92 (0.35) |  | 1.67 (0.07) | 1.12 (0.08) |
| DC of nitrogen, % |  |  |  |  |  |  |  |  |
| Microbiota variance | 3.22 (0.19) | 3.02 (0.17) |  | - | - |  | 3.02 (0.40) | 2.77 (0.37) |
| Genetic variance | - | - |  | 1.64 (0.45) | 1.73 (0.49) |  | 0.76 (0.21) | 0.62 (0.17) |
| Residual variance | 2.08 (0.08) | 1.49 (0.11) |  | 4.64 (0.44) | 3.69 (0.45) |  | 1.58 (0.27) | 1.17 (0.23) |

FCR = feed conversion ratio; DFI = daily feed intake; ADG = average daily gain; RFI = residual feed intake; DC = digestibility coefficient

**Table S4 Rank correlations of estimated microbiota values (EMV) between the model with only microbiota and the model with genetic and microbiota for feed and digestive efficiency traits records in the conventional diet and in the high fiber diet, along with their 95% confidence intervals (CI)**

|  | **CO diet** | | |  | **HF diet** | | |
| --- | --- | --- | --- | --- | --- | --- | --- |
| **Item** | **Spearman correlation** | **95% CI** | |  | **Spearman correlation** | **95% CI** | |
|  |  | **Lower** | **Upper** |  |  | **Lower** | **Upper** |
| Feed efficiency and growth traits |  |  |  |  |  |  |  |
| EMV FCR | 0.98 | 0.98 | 0.99 |  | 0.98 | 0.98 | 0.99 |
| EMV DFI | 0.98 | 0.97 | 0.98 |  | 0.98 | 0.98 | 0.99 |
| EMV ADG | 0.98 | 0.97 | 0.98 |  | 0.96 | 0.95 | 0.97 |
| EMV RFI | 0.99 | 0.98 | 0.99 |  | 0.99 | 0.98 | 0.99 |
| Digestive efficiency traits |  |  |  |  |  |  |  |
| EMV DC of energy | 0.99 | 0.99 | 1.00 |  | 0.99 | 0.99 | 1.00 |
| EMV DC of organic matter | 0.99 | 0.99 | 1.00 |  | 0.99 | 0.99 | 1.00 |
| EMV DC of nitrogen | 0.99 | 0.99 | 1.00 |  | 0.99 | 0.99 | 1.00 |

FCR = feed conversion ratio; DFI = daily feed intake; ADG = average daily gain; RFI = residual feed intake; DC = digestibility coefficient
