# Supplementary material for: Gut microbiota and host genetics contribute to the phenotypic variation of digestive and feed efficiency traits in growing pigs fed a conventional and a high fiber diet
Source: Genet Sel Evol. 2022 Jul 27;54:55. doi: 10.1186/s12711-022-00742-6 (PMC9327178; doi:10.1186/s12711-022-00742-6)
Supplement: Supplementary file 2 — Additional file 2: Script S1. Script to implement the genetic-by-diet interaction model—example for ADG. Detailed script for the example of the G × D interaction for ADG. [file 12711_2022_742_MOESM2_ESM.docx]

**Additional file 2**

**Script S1 Script to implement the Genetic by Diet Interaction Model – Example for ADG**

rm(list = ls())

library('BGLR')

perf <-

read.table("perf_animals.txt", sep = "\t") #importation of performances of animals

G <-

read.table("genomic_relationship_matrix.txt",

sep = "\t",

header = TRUE) #importation genomic relationship matrix

G <- as.matrix(G)

# Preparation of ADG vector according to the diet

ADG <- perf$ADG

phenotype <- perf

phenotype$CO <- phenotype$ADG

phenotype$HF <- phenotype$ADG

for (i in 1:nrow(phenotype)) {

if (phenotype[i, 10] == "CO")

phenotype[i, 26] <- phenotype[i, 19]

else

phenotype[i, 26] <- NA

}

for (i in 1:nrow(phenotype)) {

if (phenotype[i, 10] == "FD")

phenotype[i, 27] <- phenotype[i, 19]

else

phenotype[i, 27] <- NA

}

phenotype <- as.matrix(phenotype)

ADG <- as.vector(phenotype[, c(26, 27)])

ADG <- as.numeric(ADG)

# Preparation of fixed effects

# 1- effect of the penxbatch

perf$penxbatch <- paste(perf$batch, perf$pen, sep = "")

perf$penxbatch <- as.character(perf$penxbatch)

perf$penxbatch[is.na(perf$penxbatch)] <-

"X" #creation of extra level with all the missing values (level=X)

perf$penxbatch <- as.factor(perf$penxbatch)

W <- IncMat(perf$penxbatch)

W1 <- IncMat(perf$penxbatch)

W2 <- rbind(W, W1)

#2 – effect of the weight at the start of control

perf$weight_start_ctl[is.na(perf$weight_start_ctl)] <-

mean(perf$weight_start_ctl, na.rm = T) #remplacing missing values by the mean

X3 <- as.matrix(perf$weight_start_ctl)

X4 <- rbind(X3, X3)

# Main effects of markers

G0 <- kronecker(matrix(nrow = nEnv, ncol = nEnv, 1), G)

# Adding interaction terms

tmp <- rep(0, 2)

tmp[1] <- 1

G1 <- kronecker(diag(tmp), G)

tmp <- rep(0, 2)

tmp[2] <- 1

G2 <- kronecker(diag(tmp), G)

# Model Fitting

ETA = list(

weight = list(X = X4, model = "FIXED"),

penxbatch = list(X = W2[, -1], model = "FIXED"),

geno = list(

K = G0,

model = "RKHS",

saveEffects = TRUE

),

geno_CO = list(

K = G1,

model = "RKHS",

saveEffects = TRUE

),

geno_HF = list(

K = G2,

model = "RKHS",

saveEffects = TRUE

)

)

GxD_ADG <-

BGLR(

y = ADG,

ETA = ETA,

nIter = 120000,

burnIn = 20000,

thin = 20,

verbose = F

)
